# Identifying Mood Instability and Circadian Rest‐Activity Patterns Using Digital Remote Monitoring and Actigraphy in Participants at Risk for Bipolar Disorder

**DOI:** 10.1111/bdi.70156

**Published:** 2026-07-16

**Authors:** Priyanka Panchal, Natalie Nelissen, Niall M. McGowan, Lauren Z. Atkinson, Kate E. A. Saunders, Paul J. Harrison, Matthew Rushworth, Dejan Draschkow, John Geddes, Anna C. Nobre, Catherine J. Harmer

**Affiliations:** ^1^ Department of Psychiatry University of Oxford, Warneford Hospital Oxford UK; ^2^ DWP Digital Leeds UK; ^3^ Oxford Health NHS Foundation Trust, Warneford Hospital Oxford UK; ^4^ Department of Experimental Psychology University of Oxford Oxford UK

**Keywords:** affective instability, digital technology, mood instability, remote monitoring, rest‐activity patterns

## Abstract

**Introduction:**

Mood and/or affective instability and circadian rhythm disruptions are increasingly recognised in psychiatric disorders, notably bipolar disorder (BD), but their interrelationship remains unclear. By definition, both have an integral temporal component and, as such, measuring them longitudinally and remotely is desirable.

**Methods:**

We assessed the feasibility and value of digital devices to capture mood and subjective state (affect) instability and daily rest‐activity patterns over a 10‐week period in two groups of participants. The first group (*n* = 37) scored > 7 on the Mood Disorder Questionnaire (MDQ) (‘high MDQ’), indicating a history of mood elevation and increased risk for BD. They were compared with a group (*n* = 37) scoring < 5 on the MDQ (‘low MDQ’). Over the 10‐week period, mood was rated daily, clinical ratings of depression, mania and anxiety were captured weekly, and a GENEActiv actigraph was worn to collect rest‐activity pattern data.

**Results:**

The main findings were that (1) MDQ score predicted instability in mood and subjective state; (2) high MDQ score was associated with greater negative affect, mood symptoms and altered circadian activity profiles compared with low MDQ; and (3) mood and subjective state instability appeared unrelated to circadian indices.

**Conclusion:**

These findings suggest that (1) remote monitoring of these domains is feasible and valuable; (2) selection of participants based on MDQ score is useful for studying mood and affective instability; and (3) this approach has potential utility for clinical and experimental medicine studies assessing interventions targeting mood dysregulation and affective instability.

## 1 Introduction

Mood and/or affective instability refers to oscillations in mood and subjective state (or affect), with difficulty in regulating their behavioural consequences. These instabilities have increasingly been recognised as important dimensional features of bipolar disorder (BD), including outside of acute mood episodes. Disruption in circadian rest‐activity patterns is often seen alongside these mood and subjective state changes, but it is not known whether these observations are linked. These patterns refer to the timing, regularity and amplitude of sleep–wake and daytime activity rhythms across the 24‐h cycle, commonly measured using actigraphy‐derived indices. Combined, these affective, mood and circadian rest‐activity processes may therefore provide important mechanistic information about vulnerability to mood episodes in BD and represent potential targets for treatment.

Mood instability has been highlighted as a risk factor for the development of BD and is associated with poorer outcomes [1, 2]. However, it is often measured retrospectively [3], which can introduce biases in recall due to current mood state or other factors affecting memory. Improving how we monitor, assess and define affective and mood instability is critical for our mechanistic understanding and its potential as a treatment target in BD. These instabilities have been conceptualised and operationalised using several related but distinct statistical approaches, with instability metrics based on successive differences, such as the root mean square of successive differences (RMSSD) and *timed* root mean square of successive differences (*t*RMSSD), considered particularly sensitive to temporal fluctuations in affective states [4, 5]. Recent ecological momentary assessment and smartphone‐based studies have demonstrated that individuals across the bipolar spectrum show altered affective dynamics, including greater instability, variability and reactivity of affective states across multiple timescales [6, 7]. The emergence of new technologies which enable the collection of digital data through smartphones, tablets and wearable devices offers a sensitive avenue for the prospective monitoring of symptoms over time, aligning with broader approaches in digital phenotyping and remote behavioural assessment in psychiatry [8, 9].

Circadian rest and activity pattern differences are also commonly associated with BD. Meta‐analytic evidence suggests that sleep–wake disruption persists even during euthymic periods in bipolar disorder and may represent a trait‐like marker of illness vulnerability [10, 11]. Sleep problems are common [12, 13], where insomnia or hypersomnia is associated with periods of depression and a reduced need for sleep is associated with periods of mania [14]. These disruptions are known to persist in euthymia and periods of remission, and have been shown to encompass associated reductions in daytime activity [15, 16] and greater fragmentation of daily rest‐activity patterns [17, 18, 19]. Limiting circadian rhythm disturbance may be important in bipolar disorder given evidence for a link between this disturbance and subsequent mood episode [20, 21]. Stabilising circadian rhythm disruptions has therefore been recommended in clinical guidelines [22, 23].

This proposed interlinked relationship has further been shown across the spectrum of mood symptoms, that is, outside of acute mood episodes. For instance, individuals reporting periods of mood elevation and those showing depression and mania symptoms (thus, a BD phenotype) have been shown to display particularly lower objective circadian relative amplitude—a differentiation marker of daytime and night‐time activity—driven by increased movement or activity during rest periods, compared to those without a BD phenotype [13, 24, 25]. These disruptions, measured using actigraphy and over periods of 1–2 weeks, remained present when individuals who went on to meet diagnostic criteria for BD were excluded [13], suggesting the significance of this disruption with the experience of mood symptoms outside of particular functional impairment. Furthermore, these patterns showed modulations with mood such that those with a history of depression had the lowest circadian amplitude and variance in this marker was more strongly associated with susceptibility to mania rather than depression [24].

These findings suggest a relationship between mood symptoms and disruption in circadian rest‐activity patterns outside of specific diagnostic categories, but whether this co‐varying relationship exists longitudinally remains unclear. Recent smartphone and actigraphy studies have reported associations between mood instability, activity instability and sleep variability in individuals with BD and bipolar spectrum psychopathology [26, 27, 28, 29]. However, findings across studies remain heterogeneous, potentially reflecting differences in illness stage, symptom severity, monitoring duration and the specific actigraphy metrics used [30]. Longitudinal investigations emphasising temporal sensitivity in both mood and affective instability and circadian rest‐activity patterns may therefore aid mechanistic understanding of vulnerability to mood disorders and help identify targets for future interventions.

In this study, we tested the interrelationship between subjective state instability, mood instability and circadian rhythm disruption across a 10‐week period in a group of individuals at low and high risk for BD (assessed by the Mood Disorder Questionnaire (MDQ) [31]). This approach allows us to explore underlying processes involved in mood disturbance in the absence of medication confounds, which can affect both mood and circadian rhythm instability.

We used a similar approach to Rock et al. [13], to identify individuals with experience of mood elevation, as assessed by the MDQ. We compared those with and without self‐reported mood elevation on ratings of subjective state and mood symptoms assessed across different timescales (daily vs. weekly) using digital tablet‐based assessments alongside actigraphy. This design enabled a finer‐grained and prospective assessment of subjective state instability, mood instability and circadian rest‐activity patterns than is typically provided by retrospective reports.

By focusing on the ability of remote and wearable digital technologies to provide high‐quality and temporally sensitive data, we hypothesised that participants scoring highly on the MDQ would show a higher level of instability both for positive and negative affect. We also expected the high MDQ group to show higher ratings of negative affect and higher levels of manic and depressive symptoms, consistent with previous research [32]. Furthermore, based on prior findings in BD and bipolar spectrum phenotypes, we predicted that the high MDQ group would show specific disruptions in circadian rest‐activity patterns, including lower daytime activity, reduced circadian relative amplitude and greater instability in rest‐activity timing and amplitude across time. Finally, we hypothesised that greater disruption in rest‐activity patterns would be associated with longitudinal measures of subjective state and mood instability, highlighting a potential functional relationship between state, mood and circadian rhythm disturbance.

## 2 Methods

### 2.1 Study Design

The COMET study was a prospective, between‐subjects experimental cohort study over 10 weeks. All daily and weekly assessments were conducted electronically and remotely using a specialised study testing website and the True Colours online platform (www.truecolours.nhs.uk; True Colours, Department of Psychiatry, University of Oxford, Oxford, UK) on iPads (Apple Inc.) for mood assessments. Wrist‐worn GENEActiv actigraphs (ActivInsights Ltd., UK) collected actigraphy data. Data collection ran from 2015 to 2017. The study was approved by the Central University Research Ethics Committee of the University of Oxford (MSD‐IDREC‐C2‐2014‐023).

### 2.2 Participants

Participants were recruited by University of Oxford or community‐wide advertisements. Seventy‐four participants (48 female, 26 male; aged 18–49 years), selected based on MDQ [31] score, gave their written informed consent to participate in the COMET study. Thirty‐seven participants showed signs of mood elevation as measured by the MDQ (‘high MDQ’ group; MDQ ≥ 7) and 37 were age and gender matched, with MDQ ≤ 5 (‘low MDQ’ group). In order to facilitate participant matching, those who expressed interest and met criteria for the low MDQ group were entered into a database to be invited for participation once they were matched to a high MDQ participant.

We did not include the MDQ measure of functional impairment in this categorisation. We followed the approach used in Rock et al. [13], which employed the MDQ as a screening instrument to identify individuals experiencing a spectrum of mood‐related symptoms without necessarily meeting full diagnostic criteria. We adopted the same approach here to capture individuals experiencing affective instability while minimising potential confounds associated with medication use or clinical treatment. Although this broader MDQ approach may reduce diagnostic specificity, it allows investigation of dimensional mood‐related traits relevant to bipolar spectrum psychopathology.

After registering interest in the study, volunteers were electronically screened using the MDQ and several questions pertaining to demographics and current or past psychiatric health. They were excluded if they did not meet criteria for the high MDQ or low MDQ groups, had a current and/or lifetime diagnosis of a DSM‐IV axis I psychiatric illness (excluding BD I or II, MDD and anxiety disorder in the high MDQ group only); had a current or lifetime diagnosis of a neurological condition; and had a first degree relative with BD in the low MDQ group only. Exclusion criteria also included currently using (or having done so in the past 6 weeks) lithium, antidepressant, antipsychotic or anticonvulsant medication, or current substance abuse. We excluded medication use as this could affect the outcome measures and operate as a confounding factor. The DSM‐IV SCID I was used to assess all participants for current or prior psychiatric disorder at their baseline visit.

### 2.3 Mood and Subjective State Measurements

#### 2.3.1 Daily Subjective State Assessments

Participants completed daily subjective state assessments 5 days/week during the 10‐week study period, using the International Positive and Negative Affect Scale, Short Form (I‐PANAS‐SF) [33]. Here, they were required to rate the extent to which they experienced 10 emotions on a 5‐point Likert scale ranging from ‘very slightly/not at all’ to ‘extremely’. Participants were asked to answer this in relation to the last 24 h, or since they had last completed the questionnaire. The I‐PANAS‐SF scale measured positive affect using the words ‘alert’, ‘attentive’, ‘active’, ‘determined’ and ‘inspired’, and negative affect using ‘afraid’, ‘ashamed’, ‘hostile’, ‘nervous’ and ‘upset’.

#### 2.3.2 Weekly Mood Assessments

Participants were also required to answer a set of three self‐monitoring questionnaires as part of the online True Colours system (True Colours Team, Department of Psychiatry, University of Oxford) [34], once a week for the 10‐week study period. These questionnaires assessed depression using the standardised clinical scale QIDS (Quick Inventory of Depressive Symptomatology) [35], mood elevation using the clinical scale ASRM (Altman Self‐Rating Mania scale) [36] and anxiety using the GAD‐7 measure (Generalised Anxiety Disorder questionnaire) [37].

### 2.4 Circadian Rest‐Activity Measurements

#### 2.4.1 Actigraphy Device

All participants were equipped with GENEActiv Original actigraphs (ActivInsights Ltd., UK), which they wore consecutively for 28 days at a time on their non‐dominant wrist. To account for the 10‐week study period, three watches were required to span the duration of the study. In order to facilitate battery life for each 28‐day period, actigraph sampling frequency was set to 25 Hz.

#### 2.4.2 Pre‐Processing of Actigraphy Data

Data were pre‐processed using the *GGIR* package [38] for R version 3.4.2 (R Core Team, Vienna). Non‐parametric circadian rhythm analysis (NPCRA) variables [39], reflecting the structure of rest‐activity patterns, were calculated using the *GGIR* algorithm. Parameters included a marker for the average phase onset and activity levels for the 5‐h period of lowest activity in a given 24‐h period (*L*_5_), the 10‐h period of greatest activity in a given 24‐h period (*M*_10_), a marker for the consistency of rest‐activity patterns between days (IS or interdaily stability), and a marker for within‐day consolidation of rest‐activity states (IV or intradaily variability). A marker of amplitude strength, measuring the difference in activity between the most active 10‐h period (*M*_10_) and least active 5‐h period (*L*_5_), was also calculated and referred to as relative amplitude (RA). The equation for RA is given below.

$$\mathrm{RA}=\frac{M_{10}-L_{5}}{M_{10}+L_{5}}$$

Within‐subject variability of daily activity and timing (*L*_5_, *M*_10_) and RA was assessed using the *timed* root mean square of successive differences (*t*RMSSD) of daily measures. Further details of the actigraph device and pre‐processing methods are provided in Appendix S1.

### 2.5 Statistical Analysis

#### 2.5.1 *Timed* Root Mean Square of Successive Differences (*t*RMSSD)

The root mean square of successive differences (RMSSD) [40] has been suggested as a measure of instability that takes into account observation‐to‐observation differences, as opposed to overall trial variation as measured by the standard deviation. In this sense, it takes into account gradual shifts in the mean and is particularly sensitive to temporal, or time‐based, recordings. Whilst traditionally a measure used in calculating heart rate variability due to its sensitivity to high‐frequency heart fluctuations [41], it can be particularly appropriate in the study of stability in subjective state and mood due to its time domain nature and sensitivity in detecting trial‐by‐trial fluctuations.

The equation for RMSSD is given here:

$$\text{RMSSD}=\sqrt{\frac{1}{N}\;\sum_{i=1}^{N-1}{\left(x_{i+1}-x_{i}\right)}^{2}}$$

where *N* equals the number of recordings or intervals (in this case, a maximum of 51 for mood), and *x* is the value of one recording (or mood or actigraphy score). This definition, however, assumes that recordings are equally spaced in time (e.g., one recording every day at the exact same time). This is not the case with the mood data collected here wherein the five samples recorded weekly varied in time of day and consecutive intervals. Using the formula above with such variable time differences may result in artefactually high or low values of RMSSD. To address this, we use the *timed* RMSSD (*t*RMSSD) [42], defined as follows:

$$t\text{RMSSD}=\sqrt{\frac{1}{N}\;\sum_{i=1}^{N-1}{\left(\frac{x_{i+1}-x_{i}}{t_{i+1}-t_{i}}\right)}^{2}}$$

where *t*_*i*_ corresponds to the time at which sample *x*_*i*_ was recorded. For these data, the *t*RMSSD is thus obtained by first calculating each successive difference in mood (positive affect and negative affect) over each successive time difference between recordings. This value is then squared, and the result is averaged before the square root of the total is calculated [43].

#### 2.5.2 The Effect of Group and Time on Subjective State, Mood and Circadian Rest‐Activity Patterns

General linear models (GLMs) were conducted to analyse the effect of group (high MDQ or low MDQ; the between‐subjects factor), time (week; the within‐subjects factor), and group‐by‐time interaction for I‐PANAS‐SF, True Colours and actigraphy data. Separate models were conducted for weekly average and weekly instability (as measured by the *t*RMSSD) in I‐PANAS‐SF data, within the domains of positive affect and negative affect, and actigraphy data, within the rest‐activity domains of *L*_5_, *M*_10_ and RA. Given that IS and IV are overall markers only and thus, daily/weekly and instability data are not available, *t*‐tests were carried out to test for overall group differences in these markers. Individual GLMs were conducted for the QIDS, ASRM and GAD‐7 data, with additional post hoc *t*‐tests to test for group differences in instability (*t*RMSSD) scores. Greenhouse–Geisser corrected *p*‐values are reported where appropriate, and time was *z*‐scored in order to meet the assumptions of LMM analysis for actigraphy analyses. Analyses were carried out using repeated measures analyses of variance (ANOVAs) and individual sample *t*‐tests in SPSS and the *lme4* package (*t*‐tests using Satterthwaite's method) (version 1.‐17) [44] in R.

#### 2.5.3 Relationship Between Daily Subjective State Monitoring, Weekly Mood Monitoring and Circadian Rest‐Activity Patterns

Linear mixed‐effects models (LMMs) were conducted to analyse the effect of group on the relationship between daily I‐PANAS‐SF (subjective state), weekly True Colours (mood) and any significant daily circadian rest‐activity patterns found, across the 10‐week study period, using the *lme4* package (version 1.‐17) [44] in R. Here, affect, mood and time were *z*‐score transformed in order to meet the assumptions of LMM analysis. Both of these analyses were run on average and instability (as measured by *t*RMSSD) measures of subjective state, weekly True Colours mood responses and actigraphy measures, in order for the relationship between mood symptomatology, affect experiences and instability to be explored.

## 3 Results

### 3.1 Participants

Seventy‐four participants completed the COMET study and are included in analyses of mood data (high MDQ *n* = 37, low MDQ *n* = 37). Only those who had provided at least 50 days of actigraphy data, corresponding to the maximum of 50 days of mood data collected, are included in actigraphy analyses. Therefore, 62 participants are included in actigraphy analyses (high MDQ *n* = 31, low MDQ *n* = 31).

### 3.2 Demographics

Overall demographic characteristics are shown in Table 1. The high MDQ group and low MDQ group were matched for age and gender. Nine participants in the high MDQ group endorsed sufficient criteria to receive a DSM‐IV diagnosis, with a number of comorbidities.

**TABLE 1:** Demographics and descriptive statistics of the high MDQ and low MDQ groups.

		High MDQ group	Low MDQ group
Age, mean (SD)		24.65 (7.02)	25.00 (6.61)
Gender (m:f)		13:24	13:24
Diagnosis (*n*)	Bipolar II disorder	2	
	Bipolar NOS	2	
	Major depressive disorder	6	
	PTSD	1	
	Past alcohol dependence	2	

Mood data		High MDQ group (*n* = 37)	Low MDQ group (*n* = 37)
Positive affect (I‐PANAS‐SF), mean (SD)	Average	6.74 (2.74)	6.21 (3.97)
	Instability (*t*RMSSD)	4.0e‐5 (1.5e‐5)	3.4e‐5 (2.0e‐5)
Negative affect (I‐PANAS‐SF), mean (SD)	Average	2.75 (2.21)	1.14 (1.54)
	Instability (*t*RMSSD)	2.8e‐5 (1.5e‐5)	1.6e‐5 (1.4e‐5)
QIDS (True Colours), mean (SD)	Average	5.34 (2.97)	1.73 (1.44)
	Instability (*t*RMSSD)	0.41 (0.25)	0.21 (0.17)
ASRM (True Colours), mean (SD)	Average	2.30 (1.83)	0.41 (0.72)
	Instability (*t*RMSSD)	0.31 (0.18)	0.09 (0.10)
GAD‐7 (True Colours), mean (SD)	Average	3.82 (3.11)	0.96 (1.20)
	Instability (*t*RMSSD)	0.40 (0.24)	0.16 (0.12)

Actigraphy data		High MDQ group (*n* = 31)	Low MDQ group (*n* = 31)
*L*_5_, mean (SD)	Onset (hh:mm) (average)	02:13 (1.46)	02:26 (2.40)
	Onset instability (*t*RMSSD)	8.35 (4.01)	8.29 (4.78)
	Activity (average)	6.19 (1.78)	6.16 (2.29)
	Activity instability (*t*RMSSD)	1.94 (0.23)	1.95 (1.45)
*M*_10_, mean (SD)	Onset (hh:mm)	13:20 (2.19)	12:53 (2.61)
	Onset instability (*t*RMSSD)	4.93 (0.95)	4.97 (1.06)
	Activity (average)	49.78 (12.71)	62.26 (19.74)
	Activity instability (*t*RMSSD)	31.82 (23.40)	43.21 (26.93)
RA, mean (SD)	Average	0.77 (0.07)	0.81 (0.06)
	instability (*t*RMSSD)	0.08 (0.03)	0.07 (0.03)
IS, mean (SD)		0.31 (0.09)	0.36 (0.12)
IV, mean (SD)		1.03 (0.18)	1.07 (0.22)

### 3.3 Number of Daily and Weekly Mood Recordings

There were no significant differences in the number of days of I‐PANAS‐SF (high MDQ: 47.65 ± 3.69, low MDQ: 49.05 ± 1.96; *U* = 550.00, *p* = 0.137, *r* = 0.17) recordings made between the high MDQ and low MDQ groups or in the number of weeks of True Colours recordings made between the two groups (high MDQ: 9.22 ± 1.47, low MDQ: 9.22 ± 1.16; *U* = 674.00, *p* = 0.898, *r* = 0.02).

### 3.4 Number of Actigraphy Recordings

There was no significant difference in the number of days (high MDQ: 65.94 ± 6.25, low MDQ: 65.39 ± 8.57; *t*(60) = 0.29, *p* = 0.774) or weeks (high MDQ: 9.39 ± 0.99, low MDQ: 9.39 ± 1.26; *U* = 467.00, *p* = 0.842, *r* = 0.00) of actigraphy data recorded between the groups.

### 3.5 Daily Subjective State Assessments

Overall descriptive statistics for the daily I‐PANAS‐SF mood recordings are shown in Table 1.

#### 3.5.1 Negative Affect

**FIGURE 1:**
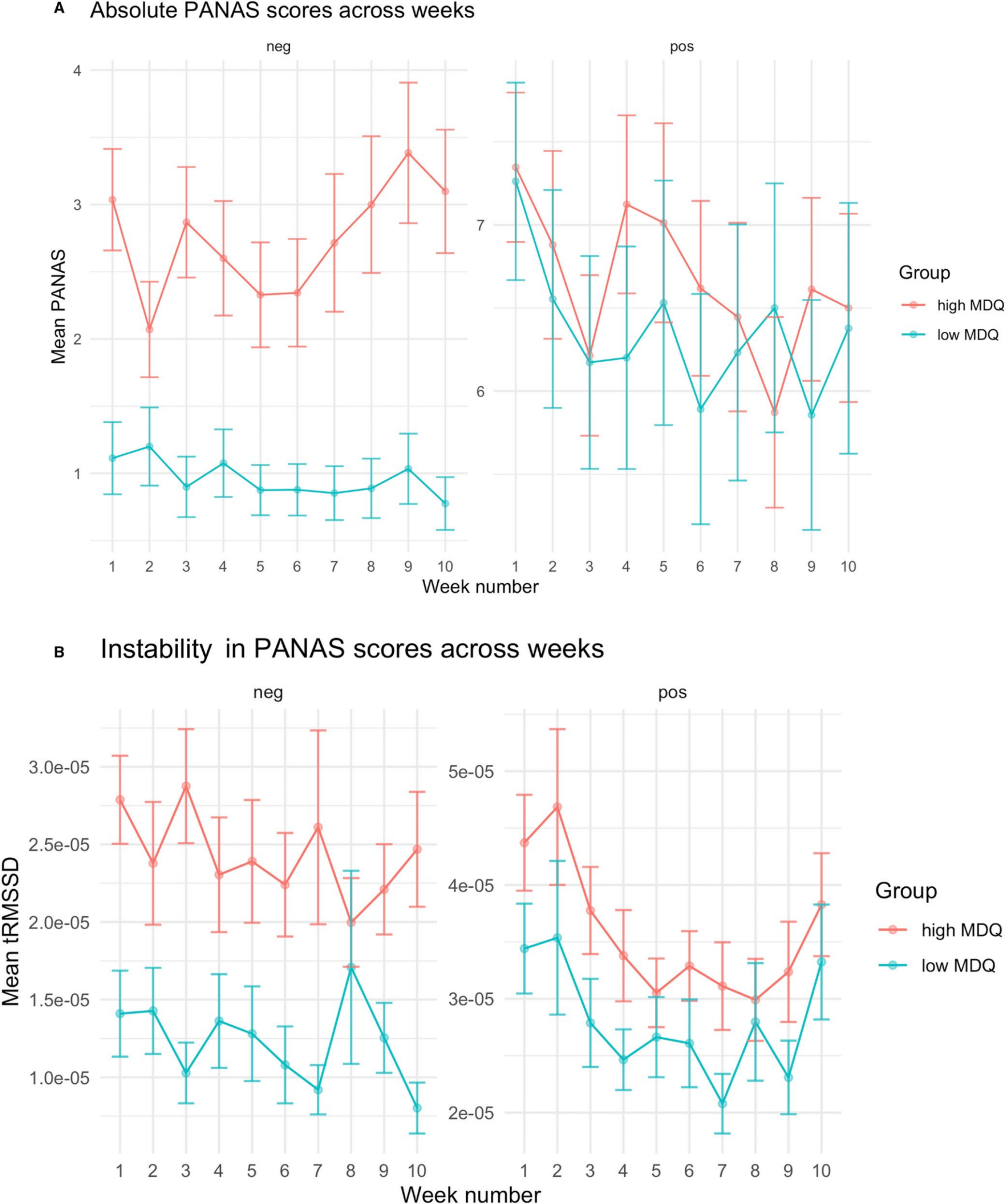
Daily I‐PANAS‐SF data across high MDQ and low MDQ groups. Panel A shows average negative affect across weeks (left) and average positive affect across weeks (right). Panel B shows instability in negative affect across weeks (left) and instability in positive affect across weeks (right), as measured by the *timed* root mean square of successive differences (*t*RMSSD). Error bars represent ±1 SEM.

The high MDQ group had greater average negative affect scores (*F*(1, 65) = 15.15, *p* < 0.001) (Figure 1A), as well as greater instability in negative affect scores as measured by *t*RMSSD (*F*(1, 61) = 14.34, *p* < 0.001) (Figure 1B). There was, however, no significant effect of time for either average or instability in negative affect (average: *F*(5.23, 339.92) = 1.72, *p* = 0.126; instability: *F*(5.54, 337.67) = 0.38, *p* = 0.882), and no group‐by‐time interaction in negative affect (average: *F*(5.54, 337.67) = 2.00, *p* = 0.076; instability: *F*(5.54, 337.67) = 0.85, *p* = 0.523).

#### 3.5.2 Positive Affect

There was no difference between groups in average positive affect scores (*F*(1, 65) = 0.05, *p* = 0.821) (Figure 1A) but the high MDQ group had greater instability in their positive affect scores compared to the low MDQ group as measured by *t*RMSSD (*F*(1, 61) = 8.18, *p* = 0.006) (Figure 1B). An effect of time was also found, such that all participants had lower positive affect ratings across the 10‐week study period (*F*(4.63, 301.21) = 2.52, *p* = 0.034), and also became less stable in their positive affect ratings across this period (*F*(5.15, 313.93) = 3.01, *p* = 0.011) but this did not interact with group (average: *F*(4.63, 301.21) = 1.04, *p* = 0.394; instability: *F*(5.15, 313.93) = 0.63, *p* = 0.679).

### 3.6 Weekly Mood Assessments

#### 3.6.1 Depressive Symptoms

**FIGURE 2:**
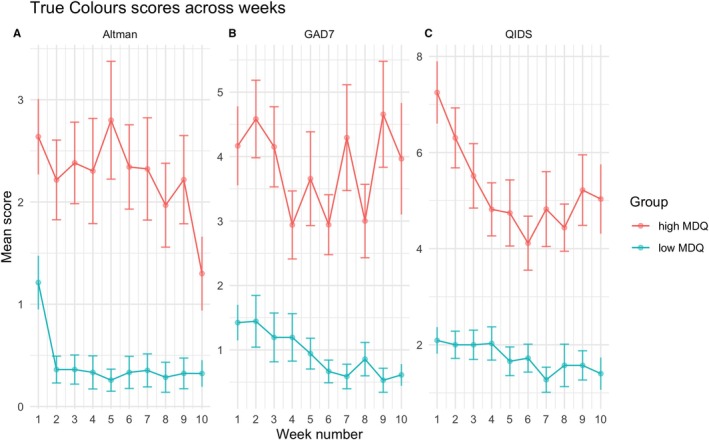
Weekly True Colours data across high MDQ and low MDQ groups. Panel A shows symptoms of mania as measured by the ASRM. Panel B shows symptoms of anxiety as measured by the GAD‐7. Panel C shows symptoms of depression as measured by the QIDS. Average scores across the 10‐week study period are shown. Error bars represent ±1 SEM.

**FIGURE 3:**
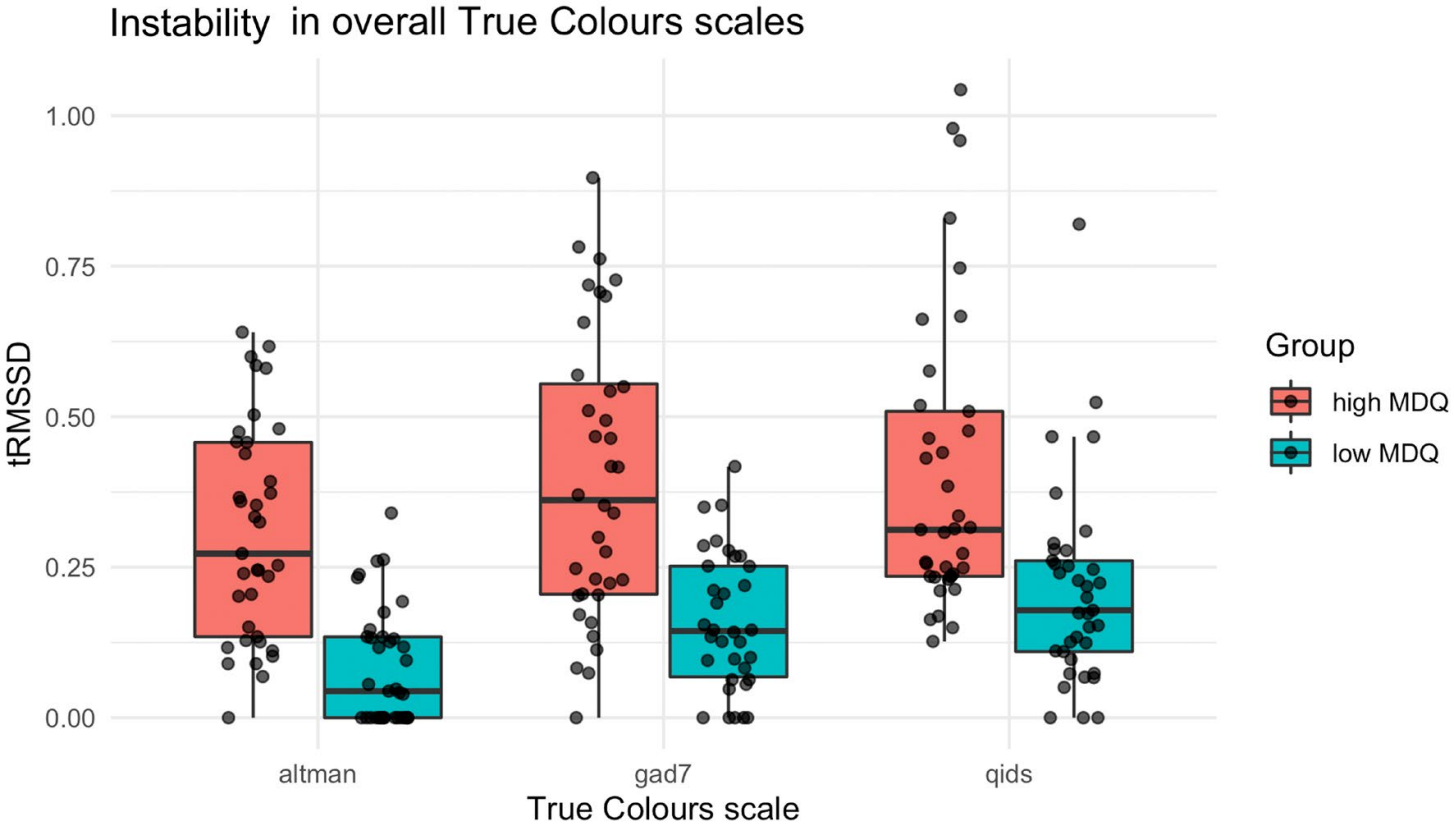
Instability in overall True Colours data as measured by the *timed* root mean square of successive differences (*t*RMSSD), across high MDQ and low MDQ groups.

Analysis of weekly QIDS data revealed that the high MDQ group had more depressive symptoms compared to the low MDQ group (*F*(1, 41) = 21.24, *p* < 0.001, see Table 1; Figure 2C). Post hoc tests on *t*RMSSD data also showed that there was more instability in these symptoms in the high MDQ group (0.41 ± 0.25) compared to the low MDQ group (0.21 ± 0.17) (*t*(71) = 3.98, *p* < 0.001) (Figure 3). Participants also reported fewer depressive symptoms across the 10‐week study period (*F*(5.69, 233.43) = 4.56, *p* < 0.001). An interaction analysis showed that this was different between the groups (*F*(5.69, 233.43) = 2.52, *p* = 0.025), such that depressive symptoms decreased in high MDQ participants to a greater degree than in low MDQ participants.

#### 3.6.2 Manic Symptoms

Analysis of weekly ASRM data revealed that the high MDQ group had more symptoms of mania compared to the low MDQ group (*F*(1, 42) = 17.11, *p* < 0.001, see Table 1; Figure 2A). Post hoc tests on *t*RMSSD data also showed that there was more instability in these symptoms in the high MDQ group (0.31 ± 0.18) compared to the low MDQ group (0.09 ± 0.10) (*t*(71) = 6.57, *p* < 0.001) (Figure 3). There was a main effect of time, such that all participants also had fewer manic symptoms over the 10‐week study period (*F*(4.94, 207.66) = 3.60, *p* = 0.004). An interaction analysis showed that this was not different between groups (*F*(4.94, 207.66) = 1.39, *p* = 0.231).

#### 3.6.3 Anxiety Symptoms

Analysis of weekly GAD‐7 data revealed that the high MDQ group had higher levels of anxiety compared to the low MDQ group (*F*(1, 41) = 16.25, *p* < 0.001, see Table 1; Figure 2B). Post hoc tests on *t*RMSSD data also showed that there was more instability in these symptoms in the high MDQ group (0.40 ± 0.24) compared to the low MDQ group (0.16 ± 0.12) (*t*(67) = 3.87, *p* < 0.001) (Figure 3). A main effect of time showed that anxiety tended to increase across the 10‐week study period (*F*(4.01, 164.25) = 2.55, *p* = 0.041). An interaction analysis showed this was not different between groups (*F*(4.01, 164.25) = 2.18, *p* = 0.073).

### 3.7 Circadian Rest‐Activity Measurements

Overall descriptive statistics for the actigraphy parameters are shown in Table 1.

**FIGURE 4:**
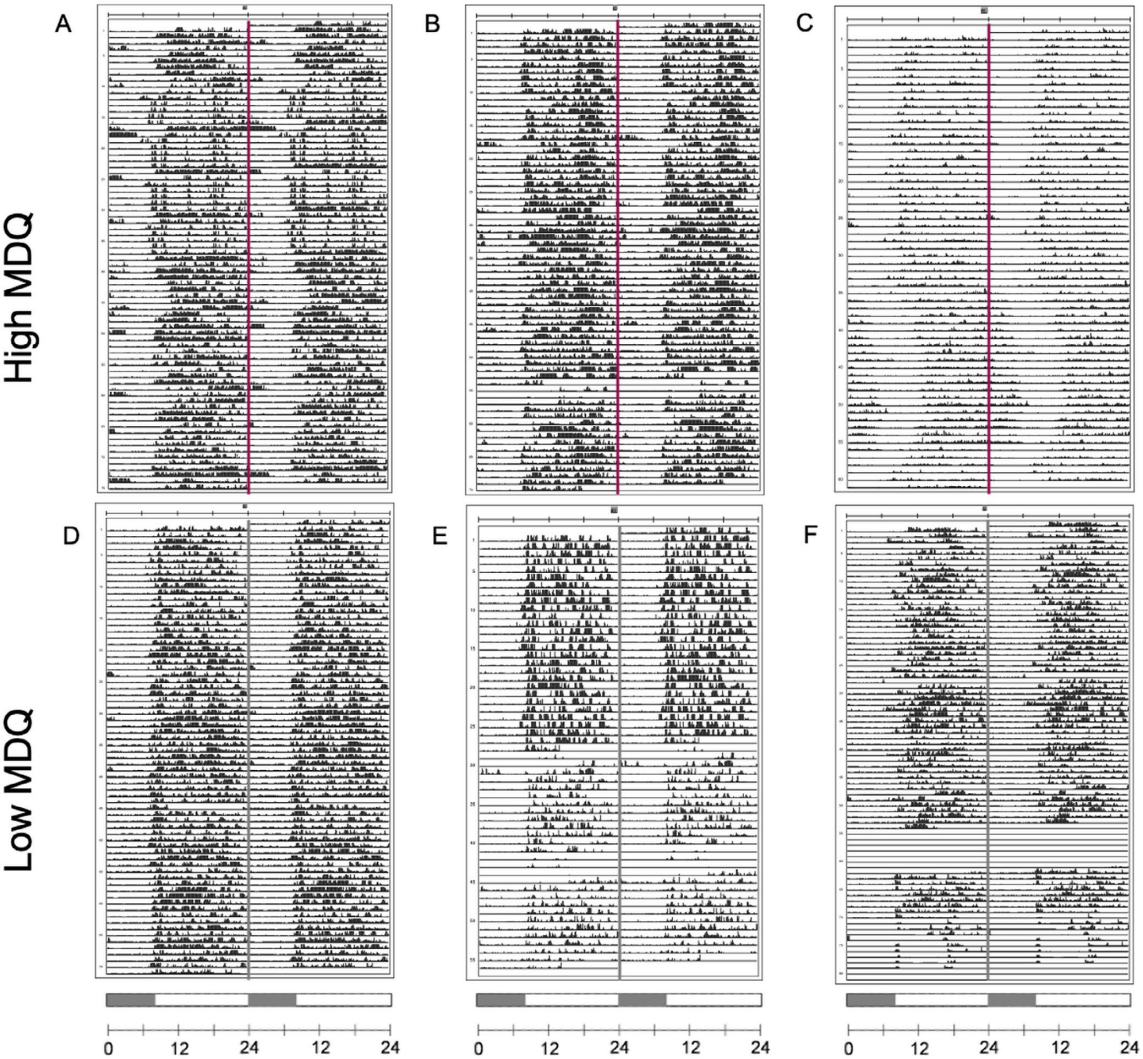
Representative actograms of rest‐activity patterns. Visual inspection of double‐plotted 24‐h actograms generated from participants' rest‐activity patterns reflects the characteristic differences detected between groups. Shaded portion of the scale bar represents the interval between 00:00 and 08:00 h as a reference guide. Actograms were generated using ActogramJ (http://actogramj.neurofly.de/).

Representative example actograms displaying 24‐h activity patterns in three high MDQ and three low MDQ participants are shown in Figure 4. Actograms from individuals in the low MDQ group (D–F) show clear distinctions in rest‐activity patterns from those of high MDQ participants (A–C).

#### 3.7.1 *L*_5_

**FIGURE 5:**
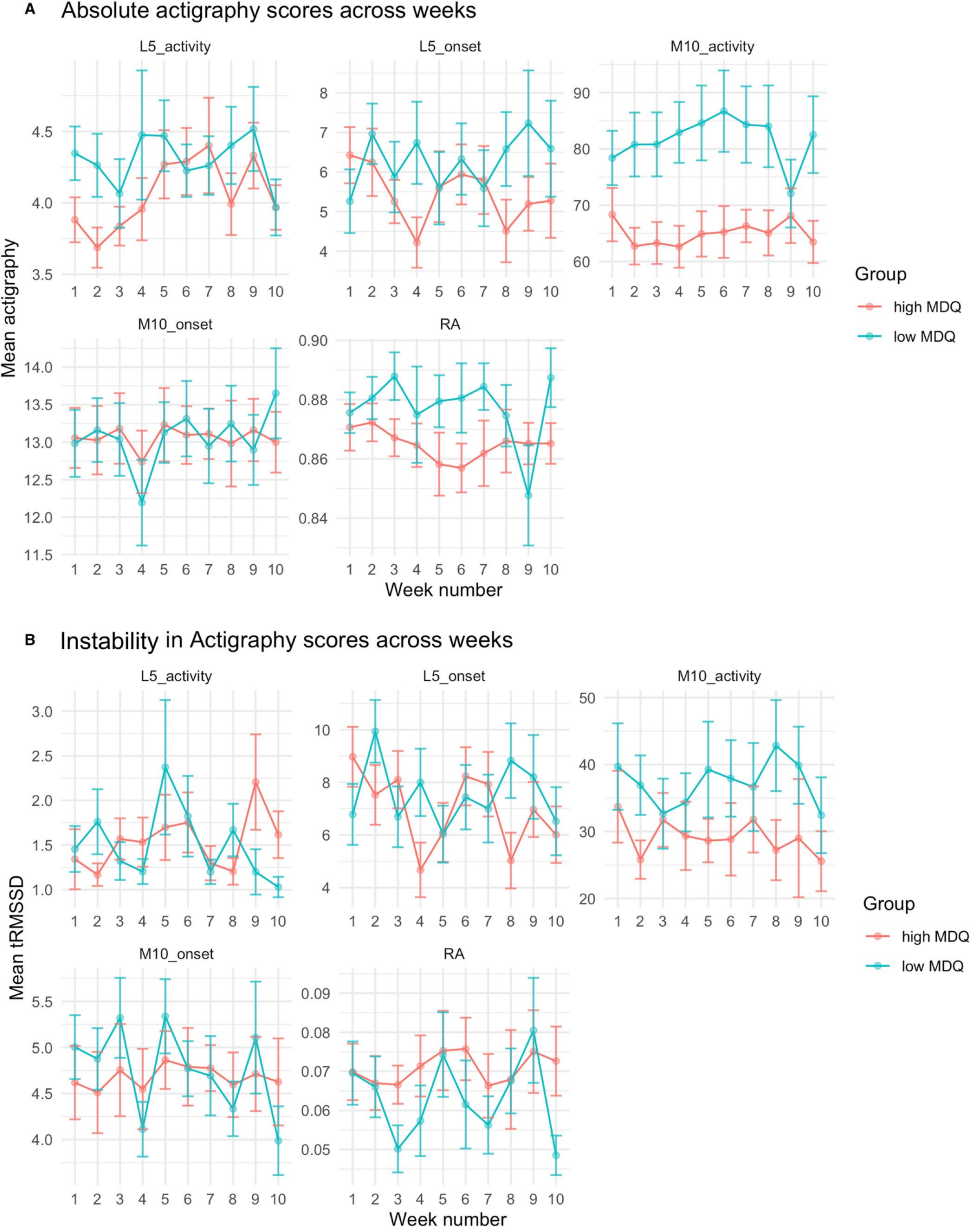
Actigraphy data across high MDQ and low MDQ groups. Panel A shows average *L*_5_ onset, *L*_5_ activity, *M*_10_ onset, *M*_10_ activity and RA across weeks. Panel B shows instability in *L*_5_ onset, *L*_5_ activity, *M*_10_ onset, *M*_10_ activity and RA across weeks, as measured by the *timed* root mean square of successive differences (*t*RMSSD). Error bars represent ±1 SEM.

There was no difference between groups in average (*t*(62.92) = 0.91, *p* = 0.364) (Figure 5A) or instability (*t*(61.75) = 0.25, *p* = 0.807) (Figure 5B) in *L*_5_ onset. There was also no effect of time on average (*t*(502.01) = −0.19, *p* = 0.846) or instability (*t*(494.64) = −1.64, *p* = 0.102) in *L*_5_ onset.

There was no difference between groups in average (*t*(61.42) = 1.37, *p* = 0.177) (Figure 5A) or instability (*t*(53.70) = 0.06, *p* = 0.950) (Figure 5B) in *L*_5_ activity. While there was no effect of time on instability (*t*(496.60) = 0.65, *p* = 0.515) in *L*_5_ activity, there was an effect of time on average (*t*(499.77) = 2.09, *p* = 0.037) *L*_5_ activity, such that *L*_5_ activity increased across the 10‐week study period.

#### 3.7.2 *M*_10_

There was no difference between groups in average (*t*(61.23) = 0.05, *p* = 0.961) (Figure 5A) or instability (*t*(61.03) = 0.46, *p* = 0.651) (Figure 5B) in *M*_10_ onset. There was also no effect of time on average (*t*(498.94) = 1.18, *p* = 0.238) or instability (*t*(503.07) = −0.71, *p* = 0.479) in *M*_10_ onset.

The high MDQ group had lower average *M*_10_ activity than the low MDQ group (*t*(61.39) = 2.84, *p* = 0.006) (Figure 5A), but there was no difference in instability in *M*_10_ activity between groups (*t*(59.61) = 1.52, *p* = 0.134) (Figure 5B). There was also no effect of time on average (*t*(495.64) = 1.69, *p* = 0.092) or instability (*t*(48.92) = 0.01, *p* = 0.995) in *M*_10_ activity.

#### 3.7.3 RA

There was no difference between groups in average (*t*(58.83) = 1.27, *p* = 0.210) (Figure 5A) or instability (*t*(52.41) = −1.35, *p* = 0.184) (Figure 5B) in RA. There was also no effect of time on average (*t*(496.05) = −1.18, *p* = 0.239) or instability (*t*(491.87) = 0.55, *p* = 0.585) in RA.

#### 3.7.4 IS and IV

**FIGURE 6:**
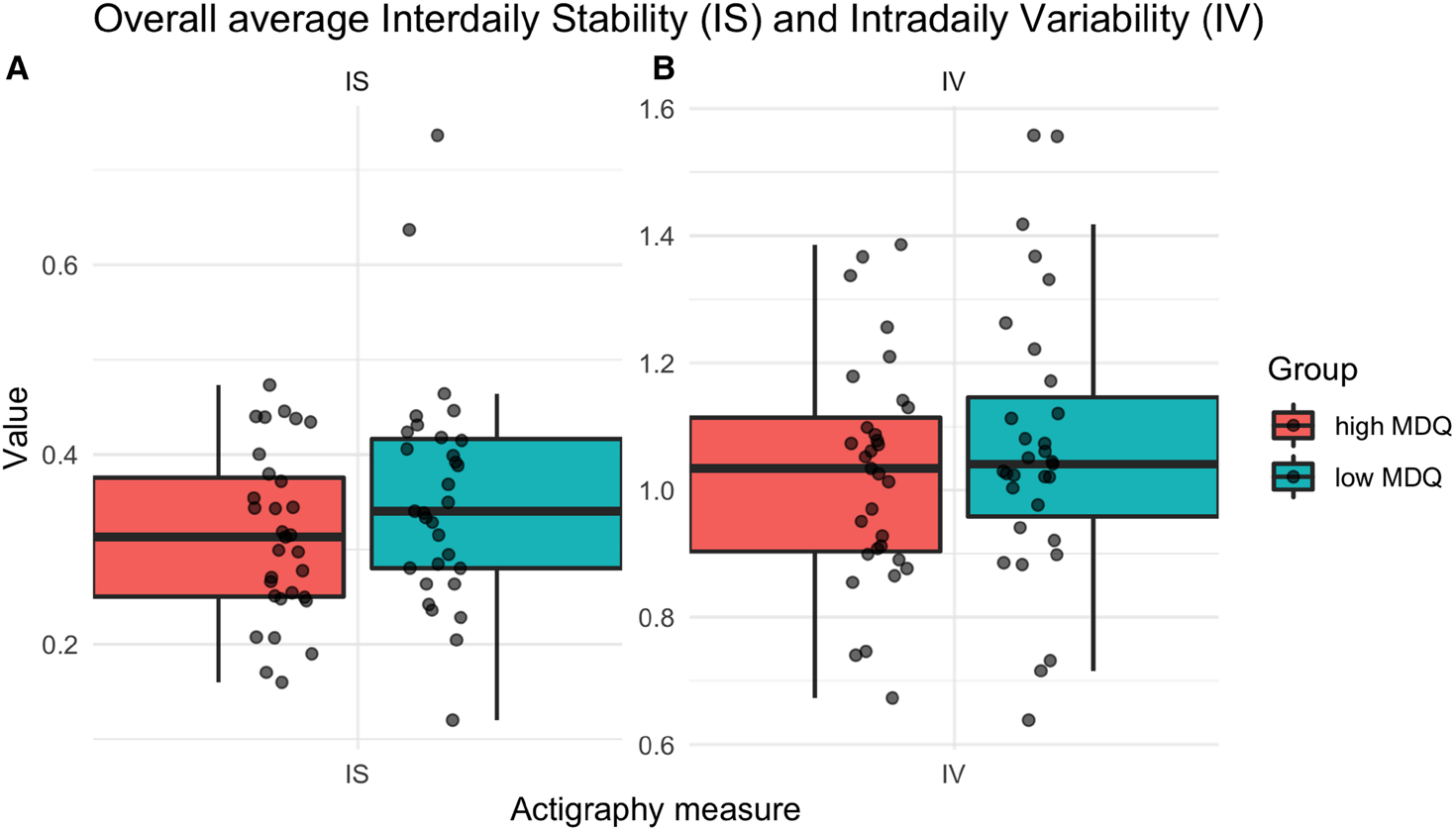
Overall interdaily stability (IS) and intradaily variability (IV) actigraphy markers, across high MDQ and low MDQ groups.

There was no difference between groups in average IS (*t*(60) = 1.59, *p* = 0.118) (Figure 6A) and average IV (*t*(60) = 0.88, *p* = 0.383) (Figure 6B).

### 3.8 Relationship Between Daily Mood Monitoring, Weekly Mood Monitoring and Circadian Rest‐Activity Patterns

Exploratory linear mixed‐effects modelling (LMM) was conducted to test for relationships between the actigraphy measure of interest (average *M*_10_ activity) and overall and instability in affect and mood (as measured by I‐PANAS‐SF and True Colours) across time. There were no significant associations between subjective state, mood and group interaction on *M*_10_ activity in these analyses (see Appendix S1 for full details).

## 4 Discussion

This study provides high‐frequency longitudinal information on the fluctuating nature of subjective state and mood‐related symptoms, supporting the use of remote and digital technologies for experimental medicine approaches to affective and mood instability associated with mood elevation. Individuals with elevated MDQ scores demonstrated greater instability in daily positive and negative affect ratings across the 10‐week study period compared with matched low MDQ participants. This was further supported by increased instability in weekly symptoms of depression, mania and anxiety in the high MDQ group. These findings are consistent with broader conceptualisations of affective lability and instability as dimensional features across the bipolar spectrum [5, 45], as well as previous daily life studies demonstrating altered affective dynamics, including greater instability and reactivity of affective states in bipolar spectrum psychopathology [6, 7]. They also align with more recent smartphone‐based monitoring studies reporting heightened mood and activity instability in bipolar disorder [27, 28]. Although the MDQ focuses primarily on mood elevation, individuals in the high MDQ group also reported significantly greater negative affect across the study period, supporting the hypothesis that elevated MDQ scores index broader dimensions of affective and mood instability associated with bipolar spectrum psychopathology.

The current findings also support the presence of disruptions in circadian rest‐activity patterns. Specifically, the high MDQ group demonstrated lower daytime activity levels, consistent with previous work reporting altered circadian activity profiles in both BD and MDD [13, 16, 46]. Prior studies have reported reduced circadian relative amplitude and greater disruption in sleep/activity timing among individuals with bipolar spectrum psychopathology [13, 24], while other research has linked sleep and activity variability with bipolar symptoms and affective instability [26, 29]. However, the specific pattern observed in the present study differed somewhat from earlier reports. Whereas previous studies have often emphasised increased night‐time activity or sleep disruption, the dominant feature in the current sample was reduced daytime activity. One possible explanation is the substantially longer monitoring duration used here relative to many previous actigraphy studies, which have typically monitored participants for fewer than 2 weeks. Extended monitoring may have enabled detection of more stable trait‐like reductions in daytime activity rather than shorter‐term or episodic sleep disturbances.

Differences in sample characteristics may also explain divergence from some prior findings. This study focused on individuals with elevated MDQ scores who were largely unmedicated and not necessarily meeting full diagnostic criteria for BD, whereas many previous studies have examined clinical BD samples. In addition to showing greater affective instability, the high MDQ group also demonstrated significantly higher negative affect and greater depressive symptoms. Together, the finding of lower daytime activity further suggests a broader depressive symptom burden in this group, consistent with literature linking psychomotor retardation, fatigue and reduced activity with depression [47]. Nevertheless, despite group differences in subjective state, mood and activity profiles, we did not observe significant longitudinal associations between concurrent subjective state instability and circadian rest‐activity measures. This contrasts with some previous smartphone and activity monitoring studies reporting dynamic associations between mood, activity and sleep instability [26, 27]. It is possible that the relatively non‐clinical nature of the present sample, differences in temporal resolution, or the specific actigraphy metrics selected contributed to these findings. More broadly, systematic reviews have highlighted substantial heterogeneity in actigraphy findings across BD studies depending on illness phase, sample characteristics and monitoring duration [30]. Future longitudinal studies in clinically diagnosed BD samples may therefore help clarify whether specific patterns of circadian disruption track changes in subjective state and mood instability over time.

## 5 Conclusion

The current study supports the use of remote and digital technologies in the diagnosis, treatment and discovery science of mood disorders, by providing high‐frequency information on the fluctuating nature of affect and mood. This is particularly important to consider given that subjective state instability and mood instability reflect a temporally sensitive, dynamic and chronic nature. Thus, the reconceptualization of symptom management—from traditional modes of in‐person clinical appointments at the temporal scale of weeks or months, to the modern use of daily and ongoing monitoring through digital technologies and passive activity sensors—is highly valuable [48]. Current findings have increased importance in their ability to show the value of low‐cost, high‐compliance, digital methods of mood monitoring in the general population, with the ability to highlight distinct changes in affect, mood and associated symptoms that may be of clinical concern.

Despite subjective state instability and mood instability not being associated with distinct differences in circadian rest‐activity rhythms, differences in these rhythms, encompassing daytime activity levels, were noted between groups. Therefore, the importance of circadian rest‐activity patterns, and the utility of evolving and remote digital technologies, cannot be ignored. Circadian patterns—of sleep, daytime activity and across the 24‐h rhythm—are increasingly considered to be core features across a range of mood disorders, including BD [49], and are known to be predictive of both onset and severity of illness [50]. Given the heterogeneous nature of these patterns, the technologies used to measure them, and the mood disorder samples studied, future research should aim to assess this putative link more stringently in order to shed light on a potential homeostatic mechanism underlying mood and associated instabilities.

It is hoped that further research to identify the neural basis of this homeostatic mechanism may help explain the unique and distinct ways in which imbalance in such regulatory systems may be associated with mood disorders. Ultimately, the identification of such mechanisms will enable the development of novel treatment targets for BD and other mood disorders, optimised at regulating subjective state and mood across a dynamic temporal scale.

## Funding

This work was supported by Wellcome Trust (102,616/Z) and National Institute for Health and Care Research (NIHR) Oxford Health Biomedical Research Centre.

## Supporting information


**Appendix S1:** Additional methodological details.
**Table S1:** Results of the linear mixed‐effects models (LMMs), testing the association between mood on average *M*_10_ activity over time. Mood by group interaction effects are also shown.

## Data Availability

The data that support the findings of this study are available from the corresponding author upon reasonable request.
