# Supplementary material for: Identifying Mood Instability and Circadian Rest‐Activity Patterns Using Digital Remote Monitoring and Actigraphy in Participants at Risk for Bipolar Disorder
Source: Bipolar Disord. 2026 Jul 16;28(5):e70156. doi: 10.1111/bdi.70156 (PMC13377006; doi:10.1111/bdi.70156)
Supplement: Supplementary file 1 — Appendix S1: Additional methodological details. Table S1: Results of the linear mixed‐effects models (LMMs), testing the association between mood on average M 10 activity over time. Mood by group interaction effects are also shown. [file BDI-28-0-s001.docx]

**Identifying mood instability and circadian rest-activity patterns using digital remote monitoring and actigraphy in participants at risk for bipolar disorder**

**Supplementary Materials**

**Methods**

Actigraphy

The actigraphs used contain a tri-axial accelerometer integrated in microelectromechanical systems and measure raw acceleration in gravitational units with a range of +/- 8g (1g = 9.8 m/s^2^). The main output measure representing participants’ moment-to-moment activity is the gravity subtracted signal vector magnitude of acceleration (Esliger et al., 2011), given by the equation:

$$Acceleration magnitude= \left( \sum\sqrt{x^{2}+ y^{2}+ z^{2}}-g \right)$$

Where *x*, *y*, and *z* are the three raw signals and 1g is subtracted to account for acceleration due to gravity. Negative values were rounded to zero, as per the Euclidean Norm Minus One (ENMO) (Hildebrand, Van Hees, Hansen, & Ekelund, 2014; Van Hees et al., 2014).

Only data included between the first and last midnight of actigraph recording were analysed for each participant. The *GGIR* non-wear algorithm ensured that any suspected actigraph removals were identified and missing intervals imputed with the average of similar time points from other full days of recording. Any days with more than three hours of missing data were excluded from further analyses (Bromundt et al., 2011; McGowan et al., 2019).

Relationship between daily mood monitoring, weekly mood monitoring, and circadian rest-activity patterns

Linear mixed-effects models (LMMs) were used to analyse the effect of group on the relationship between rest-activity patterns and mood across weeks. These analyses were conducted using the *lme4* package (version 1.-17) (Bates et al., 2015) in the R statistical programming language (R Core Team, 2022). LMMs were chosen as they allow for simultaneous estimation of between-subjects and between-recording variance, and therefore yield advantages over traditional analyses of variance (Baayen et al., 2008; Helbing et al., 2020; Kliegl et al., 2011).

The LMM was defined such that group and mood were entered as fixed effects and participant ID as the random effect, resulting in:

$$rest-activity variable \sim\left( mood \times group \right)+time+ \left( 1 | participant ID \right)$$

where the interaction between group and mood, controlling for time, was tested.

The rest-activity variable of interest, average M_10_ activity, was entered as the dependent variable, and the relationship with mood tested across all domains of the I-PANAS-SF (positive affect and negative affect) and True Colours (QIDS, ASRM, GAD-7) scales. Time and mood were *z*-scored in order to meet the assumptions of LMM analysis.

Within these weekly measures of rest-activity patterns and mood, both absolute (average as measured by the mean) and instability (as measured by *t*RMSSD) markers were explored, in line with the findings from the mood analyses. *P*-values were calculated using Satterthwaite’s degrees of freedom method via the *lmerTest* package (version 3.1-0) (Kuznetsova et al., 2017) in R (R Core Team, 2022).

**Results**

| I-PANAS-SF × M10 activity (average) | |
| --- | --- |
| Negative affect (average) | t(548.55) = 0.12, p = .901 |
| Negative affect (average) × group | t(549.08) = -0.42, p = .675 |
| Negative affect (tRMSSD) | t(513.16) = -0.86, p = .388 |
| Negative affect (tRMSSD) × group | t(513.08) = 0.55, p = .582 |
| Positive affect (tRMSSD) | t(504.09) = 0.07, p = .947 |
| Positive affect (tRMSSD) × group | t(504.08) = -0.06, p = .951 |
| True Colours × M10 activity (average) | |
| QIDS (average) | t(501.57) = -0.50, p = .615 |
| QIDS (average) × group | t(502.51) = 0.56, p = .579 |
| ASRM (average) | t(491.42) = 0.40, p = .693 |
| ASRM (average) × group | t(493.16) = 0.13, p = .900 |
| GAD-7 (average) | t(496.71) = 0.22, p = .825 |
| GAD-7 (average) × group | t(494.79) = -0.58, p = .562 |

Supplementary Table 1. Results of the linear mixed-effects models (LMMs), testing the association between mood on average M_10_ activity over time. Mood by group interaction effects are also shown.

**References**

Baayen, R. H., Davidson, D. J., & Bates, D. M. (2008). Mixed-effects modeling with crossed random effects for subjects and items. *Journal of Memory and Language*, *59*(4), 390–412. https://doi.org/10.1016/j.jml.2007.12.005

Bates, D., Mächler, M., Bolker, B. M., & Walker, S. C. (2015). Fitting linear mixed-effects models using lme4. *Journal of Statistical Software*, *67*(1). https://doi.org/10.18637/jss.v067.i01

Bromundt, V., Köster, M., Georgiev-Kill, A., Opwis, K., Wirz-Justice, A., Stoppe, G., & Cajochen, C. (2011). Sleep - Wake cycles and cognitive functioning in schizophrenia. *British Journal of Psychiatry*, *198*(4), 269–276. https://doi.org/10.1192/bjp.bp.110.078022

Esliger, D. W., Rowlands, A. V., Hurst, T. L., Catt, M., Murray, P., & Eston, R. G. (2011). Validation of the GENEA accelerometer. *Medicine and Science in Sports and Exercise*, *43*(6), 1085–1093. https://doi.org/10.1249/MSS.0b013e31820513be

Helbing, J., Draschkow, D., & Võ, M. L. H. (2020). Search superiority: Goal-directed attentional allocation creates more reliable incidental identity and location memory than explicit encoding in naturalistic virtual environments. *Cognition*, *196*. https://doi.org/10.1016/j.cognition.2019.104147

Hildebrand, M., Van Hees, V. T., Hansen, B. H., & Ekelund, U. (2014). Age group comparability of raw accelerometer output from wrist-and hip-worn monitors. *Medicine and Science in Sports and Exercise*, *46*(9), 1816–1824. https://doi.org/10.1249/MSS.0000000000000289

Kliegl, R., Wei, P., Dambacher, M., Yan, M., & Zhou, X. (2011). Experimental effects and individual differences in linear mixed models: Estimating the relationship between spatial, object, and attraction effects in visual attention. *Frontiers in Psychology*, *1*(JAN), 238. https://doi.org/10.3389/fpsyg.2010.00238

Kuznetsova, A., Brockhoff, P. B., & Christensen, R. H. B. (2017). lmerTest Package: Tests in Linear Mixed Effects Models. *Journal of Statistical Software*, *82*(13). https://doi.org/10.18637/jss.v082.i13

McGowan, N. M., Goodwin, G. M., Bilderbeck, A. C., & Saunders, K. E. A. A. (2019). Circadian rest-activity patterns in bipolar disorder and borderline personality disorder. *Translational Psychiatry*, *9*(1). https://doi.org/10.1038/s41398-019-0526-2

R Core Team. (2022). *R: A language and environment for statistical computing* (URL https://www.R-project.org/). R Foundation for Statistical Computing.

Van Hees, V. T., Fang, Z., Langford, J., Assah, F., Mohammad, A., Da Silva, I. C. M., Trenell, M. I., White, T., Wareham, N. J., & Brage, S. (2014). Autocalibration of accelerometer data for free-living physical activity assessment using local gravity and temperature: An evaluation on four continents. *Journal of Applied Physiology*, *117*(7), 738–744. https://doi.org/10.1152/japplphysiol.00421.2014
